# Supplementary material for: The inverse relationship between dietary anthocyanidins consumption and frailty: Findings from the National Health and Nutrition Examination Survey
Source: PLoS One. 2025 Aug 1;20(8):e0328489. doi: 10.1371/journal.pone.0328489 (PMC12316302; doi:10.1371/journal.pone.0328489)
Supplement: S2 Table — (DOCX) [file pone.0328489.s002.docx]

S1 Table Relationship between different anthocyanidins consumption levels and frailty after excluding participants with stroke, coronary heart disease, and depressive symptom.

|  | **Crude model** | | | **Model1** **^a^** | | | **Model2 ^b^** | | | **Model3 ^c^** | | |
| --- | --- | --- | --- | --- | --- | --- | --- | --- | --- | --- | --- | --- |
| **Characteristic** | **OR** | **95% CI** | **p-value** | **OR** | **95% CI** | **p-value** | **OR** | **95% CI** | **p-value** | **OR** | **95% CI** | **p-value** |
| **Cyanidin** *^d^* |  |  |  |  |  |  |  |  |  |  |  |  |
| group1 | — | — |  | — | — |  | — | — |  | — | — |  |
| group2 | 0.75 | 0.60, 0.93 | **0.009** | 0.58 | 0.45, 0.75 | **<0.001** | 0.64 | 0.38, 1.07 | 0.082 | 0.69 | 0.41, 1.14 | 0.14 |
| group3 | 0.81 | 0.63, 1.03 | 0.088 | 0.51 | 0.38, 0.67 | **<0.001** | 0.73 | 0.46, 1.17 | 0.2 | 0.70 | 0.45, 1.08 | 0.10 |
| **Cyanidin overall** *^e^* | 1.00 | 0.99, 1.01 | >0.9 | 0.99 | 0.97, 1.02 | 0.6 | 1.00 | 0.99, 1.01 | 0.6 | 1.00 | 1.00, 1.01 | 0.4 |
| **Malvidin** *^d^* |  |  |  |  |  |  |  |  |  |  |  |  |
| group1 | — | — |  | — | — |  | — | — |  | — | — |  |
| group2 | 0.76 | 0.62, 0.93 | **0.009** | 0.66 | 0.54, 0.81 | **<0.001** | 0.98 | 0.68, 1.42 | >0.9 | 0.99 | 0.74, 1.33 | >0.9 |
| group3 | 0.66 | 0.57, 0.78 | **<0.001** | 0.49 | 0.41, 0.57 | **<0.001** | 0.65 | 0.46, 0.90 | **0.014** | 0.63 | 0.46, 0.87 | **0.008** |
| **Malvidin overall** *^e^* | 0.99 | 0.98, 1.00 | **0.005** | 0.98 | 0.97, 0.99 | **<0.001** | 0.99 | 0.97, 1.00 | 0.058 | 0.99 | 0.98, 1.00 | 0.064 |
| **Peonidin** *^d^* |  |  |  |  |  |  |  |  |  |  |  |  |
| group1 | — | — |  | — | — |  | — | — |  | — | — |  |
| group2 | 0.94 | 0.76, 1.16 | 0.6 | 0.77 | 0.60, 0.98 | **0.036** | 0.93 | 0.57, 1.51 | 0.7 | 0.93 | 0.57, 1.49 | 0.7 |
| group3 | 0.75 | 0.63, 0.88 | **0.001** | 0.55 | 0.45, 0.67 | **<0.001** | 0.79 | 0.57, 1.09 | 0.13 | 0.75 | 0.54, 1.05 | 0.087 |
| **Peonidin overall** *^e^* | 0.99 | 0.98, 1.01 | 0.5 | 0.99 | 0.97, 1.01 | 0.4 | 1.00 | 0.98, 1.02 | >0.9 | 1.00 | 0.98, 1.02 | 0.8 |
| **Petunidin** *^d^* |  |  |  |  |  |  |  |  |  |  |  |  |
| group1 | — | — |  | — | — |  | — | — |  | — | — |  |
| group2 | 0.78 | 0.62, 0.97 | **0.029** | 0.71 | 0.55, 0.90 | **0.006** | 0.91 | 0.59, 1.39 | 0.6 | 0.90 | 0.58, 1.39 | 0.6 |
| group3 | 0.66 | 0.56, 0.77 | **<0.001** | 0.53 | 0.44, 0.63 | **<0.001** | 0.75 | 0.53, 1.05 | 0.091 | 0.72 | 0.52, 1.0 | **0.047** |
| **Petunidin overall** *^e^* | 0.98 | 0.95, 1.00 | 0.072 | 0.96 | 0.93, 0.99 | **0.007** | 0.94 | 0.90, 0.99 | **0.023** | 0.95 | 0.92, 0.99 | **0.008** |
| **Pelargonidin** *^d^* |  |  |  |  |  |  |  |  |  |  |  |  |
| group1 | — | — |  | — | — |  | — | — |  | — | — |  |
| group2 | 1.07 | 0.87, 1.32 | 0.5 | 0.89 | 0.71, 1.13 | 0.3 | 1.05 | 0.67, 1.63 | 0.8 | 1.00 | 0.67, 1.49 | >0.9 |
| group3 | 0.85 | 0.74, 0.99 | **0.035** | 0.69 | 0.58, 0.82 | **<0.001** | 1.03 | 0.74, 1.41 | 0.9 | 1.00 | 0.74, 1.36 | >0.9 |
| **Pelargonidin overall** *^e^* | 0.99 | 0.97, 1.00 | **0.045** | 0.98 | 0.96, 0.99 | **0.011** | 1.00 | 0.97, 1.02 | 0.7 | 1.00 | 0.98, 1.02 | 0.7 |
| **Delphinidin** *^d^* |  |  |  |  |  |  |  |  |  |  |  |  |
| group1 | — | — |  | — | — |  | — | — |  | — | — |  |
| group2 | 0.81 | 0.68, 0.97 | **0.022** | 0.63 | 0.52, 0.77 | **<0.001** | 0.82 | 0.58, 1.15 | 0.2 | 0.84 | 0.61, 1.14 | 0.3 |
| group3 | 0.67 | 0.57, 0.78 | **<0.001** | 0.49 | 0.42, 0.58 | **<0.001** | 0.73 | 0.52, 1.02 | 0.066 | 0.71 | 0.52, 0.97 | **0.031** |
| **Delphinidin overall** *^e^* | 0.98 | 0.96, 1.00 | **0.014** | 0.96 | 0.95, 0.98 | **0.001** | 0.96 | 0.93, 0.99 | **0.013** | 0.96 | 0.94, 0.99 | **0.006** |
| **Total Anthocyanidins** *^d^* |  |  |  |  |  |  |  |  |  |  |  |  |
| group1 | — | — |  | — | — |  | — | — |  | — | — |  |
| group2 | 0.86 | 0.69, 1.09 | 0.2 | 0.65 | 0.50, 0.85 | **0.002** | 0.73 | 0.45, 1.20 | 0.2 | 0.76 | 0.47, 1.25 | 0.3 |
| group3 | 0.64 | 0.51, 0.81 | **<0.001** | 0.40 | 0.31, 0.53 | **<0.001** | 0.60 | 0.38, 0.94 | **0.028** | 0.58 | 0.37, 0.90 | **0.018** |
| **Total Anthocyanidins overall** *^e^* | 1.00 | 0.99, 1.00 | 0.070 | 0.99 | 0.99, 1.00 | **0.007** | 1.00 | 0.99, 1.00 | 0.3 | 1.00 | 0.99, 1.00 | 0.3 |

**Abbreviations:** OR, odds ratio; CI, confidence interval.

**a** Model 1 was adjusted for age, sex (male and female) and race (Mexican American, non-Hispanic black, non-Hispanic white, other races).

**b** Model 2 was additionally adjusted for education (below high school, high school, above high school), marital status (married/living with partner, never married, divorced/separated, widowed), poverty-income ratio (continuous), body mass index（BMI） (<18.5, 18.5≤BMI<25, 25≤BMI<30, ≥30, kg/m2), drinking status (no drinker, moderate drinker, heavy drinker), smoking (never, former, current), caffeine consumption(continuous, gram/day), total dietary energy intake(continuous, kcal/day), and physical activity( low, moderate, high);

**c** Model 3 was additionally adjusted for white blood cells count (1000 cells/μL), hemoglobin (g/dL), platelets count (1000 cells/μL), albumin (g/dL), creatinine (mg/dL), uric acid (mg/dL), alanine transaminase (U/L) and aspartate transaminase (U/L), hypertension, hyperlipidemia, and diabetes.

**d** The consumption of each type of anthocyanidin was categorized into three groups: Group 1 (no consumption, i.e., intake = 0), Group 2 (intake below the median), and Group 3 (intake at or above the median).

**e**：Analysis of each anthocyanidin subtype and total anthocyanidins as continuous intake variables.
