# Supplementary material for: The inverse relationship between dietary anthocyanidins consumption and frailty: Findings from the National Health and Nutrition Examination Survey
Source: PLoS One. 2025 Aug 1;20(8):e0328489. doi: 10.1371/journal.pone.0328489 (PMC12316302; doi:10.1371/journal.pone.0328489)
Supplement: Attachment — Submitted filename: R_Comments_PONE-D-25-11030.pdf [file pone.0328489.s003.pdf]

Reviewer’s Comments

Title: The inverse relationship between dietary anthocyanidins consumption and Frailty: Findings from the National Health and Nutrition Examination Survey

Title: Satisfactory

Abstract: Satisfactory

Introduction:

- Regarding “Therefore, the aim of this study is to ……”: Since the research has already been completed, it would be more appropriate to use the past tense.
- Authors claim that this is the first to investigate the link between anthocyanidin intake and frailty in a nationally representative U.S. adult population using NHANES data. However, a study published in 2025 in Frontiers in Nutrition analyzed data from NHANES 2007–2010 and 2017–2018, and has examined the association between dietary flavonoid intake, including anthocyanidins, and frailty in U.S. adults. It would be beneficial to acknowledge and reference this prior work in the introduction to better contextualize the current study.

Materials and Methods:

- Why were only the NHANES cycles from 2007-2010 and 2017-2018 used, and not other years?
- Figure numbers and figure titles with legends have not been included, appropriately. Figures are not even placed in order, at the end of the manuscript.
- In Figure 1 (flow chart), it would be helpful to clearly mention the specific criterion/ criteria used for the initial exclusion of participants.

- Why were only the first and second days of dietary intake used for analysis?

- Regarding “To derive the frailty index value, the participant's acquired deficits' number is divided by the total potential deficits.”: Suggest to cite correct primary source (reference) that authors used for Frailty Index Calculation.

Results:

- “Among these participants, 3,338 (18%) were non-frail, while 11,182 (82%) were classified as frail.” Is this correct? Table 1 shows the opposite trends.
- Please recheck the calculated percentages presented in Table 1, as it appears discrepancies in the current values.

- In Table 1, “[1]N not Missing (unweighted), [2]Mean ± SD; n (unweighted) (%), [3]t-test adapted to complex survey samples; chi-squared test with Rao & Scott's second-order correction.” Here, numbers have not been superscripted.

- Under “Characteristics of the participants at baseline”:
    - o It says “….. lower hemoglobin and uric acid levels, ……”, however, frail individuals exhibited higher uric acid levels. Please revise it to ensure correctness.
    - o Instead of “(refer to Table 1)”, keep it as (Table 1).
- Given the large sample sizes, there is a high likelihood of obtaining a statistically significant result even for small differences. Therefore, suggest to report the effect size (such as Cohen's d) alongside the p-value to assess the practical significance of the result.
- Additionally, based on the reported standard errors, it appears that the assumption of equal variances may not hold. Suggest “Levene's test” to formally assess the equality of variances. If the variances are unequal, the Welch’s t-test, which does not assume equal variances, should be used instead of the standard Student’s t-test.
- For comparisons using the chi-square test, since the sample size is large, the assumption regarding expected frequencies is likely satisfied. However, when working with contingency tables larger than 2×2, and if the result is statistically significant, it is important to perform a standardized residual analysis. This helps to identify which specific cells (i.e., combinations of categories) contribute most to the observed association.

- Consider removing the full-stop at the end of subheadings.
- Under “Relationship between anthocyanidins consumption and frailty”: consider adding the word “respectively” in relevant sentences to clarify the order of items and improve readability.
- “Similarly, the overall relationship between different types of anthocyanidins and frailty was not significant in all models, except for delphinidin, which exhibited an OR of 0.97 (95% CI: 0.96, 0.99), 0.96 (95% CI: 0.94,0.98), 0.96 (95% CI: 0.94, 0.99), and 0.97 (95% CI: 0.94, 1.00) in the crude model, Model 1, Model 2, and Model 3, respectively.” Check this for clarity. Is this consistent with Table 2?
- In table 2, “a, b, c, and d” are not superscripted.
- In table 2, “overall c”: what “c” stands for?
- In table 2: “[c]Model 3 was additionally adjusted for white blood cells count (1000 cells/ul), …….. alanine transaminase (u/l) and aspartate transaminase, hypertension, …….., and stroke.” Here, the unit for aspartate transaminase is missing, while units are clearly stated for other parameters. Suggest to include the appropriate units for consistency and clarity.
- Under “subgroup analysis of the relationship between anthocyanidins consumption and frailty”,

  - It is mentioned that "In the 41-60 age group, an elevated intake of malvidin at or above the median correlated with a decreased frailty risk (OR = 0.769; 95% CI: 0.563-1.051)." However, OR and CI values do not appear to correspond correctly with those in Table 3. Please review and revise accordingly.
  - Regarding "Similarly, the odds ratio of frailty was 0.567 (95% CI: 0.404-0.796) for consuming less than the median of total anthocyanidins, decreasing further to 0.452 (95% CI: 0.318-0.644) in 41-60 years old adults.": Here, it appears to refer to Group 3, however, this is not clearly stated. Please clarify.
  - Regarding "Specifically, higher intake of malvidin, petunidin, and delphinidin significantly correlated with lower odds of frailty in males, with odds ratios (OR) of 0.724 (95% CI: 0.525-0.998), 0.673 (95% CI: 0.491-0.923), and 0.702 (95% CI: 0.519-0.950), respectively.": Here, is higher intake of malvidin significantly correlated with lower odds of frailty in males, as p value is 0.055? In methodology, the authors stated that the significance level was set at 0.05. Please justify the rationale.
- In table 3, what are meant by "P for trend" and "P for interaction"?
- Suggest to indicate how the p-values were generated (e.g., statistical test used) in the footnote of Table 3 for clarity.
- In table 3, "a" (at the end of group names) needs to be superscripted.
- Authors have used four logistic regression models to assess the association between anthocyanidin intake and frailty. However, a formal comparison of model fit, such as a likelihood ratio test (using ANOVA on nested models) or reporting AIC/BIC values can be performed to justify the incremental addition of covariates across models. It would strengthen the rationale for model selection and highlight whether each adjustment meaningfully improves explanatory power. Since your focus is on model-adjusted associations, demonstrating improvements in model fit could further support the robustness of your conclusions.
- Supplementary Tables have not been attached along with the manuscript.
- Under "Dose-response relationships between anthocyanidins intake and frailty risk": it says "In the investigation of ….. total anthocyanidins intake and frailty ($P < 0.01$). is the p value mentioned, correct?

Discussion:

- Fourth paragraph: ("our study exhibits …….. progression over time.") appears to be paraphrased again in the fifth paragraph.
- Suggest to incorporate findings from a similar recent study published in 2025 in Frontiers in Nutrition to enrich your discussion and provide up-to-date context.

Conclusion: Satisfactory

References:

- Review and revise the references to ensure consistency in formatting throughout the manuscript.

Overall/ common:

- Fonts and font sizes are not uniform throughout the manuscript.
