# Supplementary material for: The inverse relationship between dietary anthocyanidins consumption and frailty: Findings from the National Health and Nutrition Examination Survey
Source: PLoS One. 2025 Aug 1;20(8):e0328489. doi: 10.1371/journal.pone.0328489 (PMC12316302; doi:10.1371/journal.pone.0328489)
Supplement: Attachment — Submitted filename: Response to Reviewers.pdf [file pone.0328489.s006.pdf]

Response to editor:

Q1. Please ensure that your manuscript meets PLOS ONE's style requirements, including those for file naming. The PLOS ONE style templates can be found at

https://journals.plos.org/plosone/s/file?id=wjVg/PLOSOne_formatting_sample_main_body.pdf and

https://journals.plos.org/plosone/s/file?id=ba62/PLOSOne_formatting_sample_title_authors_affiliations.pdf

A1: We have made corresponding revisions to the article in accordance with PLOS ONE's style requirements.

Q2. This work was supported by the Medical Science and Technology Project of Zhejiang Province(No. 2022KY576,No.2023KY453).

Please provide an amended statement that declares *all* the funding or sources of support (whether external or internal to your organization) received during this study, as detailed online in our guide for authors at http://journals.plos.org/plosone/s/submit-now. Please also include the statement "There was no additional external funding received for this study." in your updated Funding Statement.

Please include your amended Funding Statement within your cover letter. We will change the online submission form on your behalf.

A2: We have amended Funding Statement:

This work was supported by the Natural Science Foundation of Zhejiang Province (No. LGF21H170005), the Medical Science and Technology Project of Zhejiang Province (No. 2022KY576, No.2023KY453), and Zhejiang Traditional Chinese Medicine Administration (No. 2024ZL269). There was no additional external funding received for this study. The funding agencies was not involved in the study design, data collection and analysis, decision to publish, or preparation of the manuscript.

Q3. Thank you for stating the following in the Acknowledgments Section of your manuscript:

The present study utilizes data sourced from the National Health and Nutrition Examination Survey (NHANES). We express our gratitude to the National Center for

Health Statistics (NCHS) and the United States Centers for Disease Control and Prevention (CDC) for their generous funding assistance in facilitating the gathering and analysis of data. We express our gratitude to both the study participants and the staff involved in this research endeavor.

We note that you have provided funding information that is not currently declared in your Funding Statement. However, funding information should not appear in the Acknowledgments section or other areas of your manuscript. We will only publish funding information present in the Funding Statement section of the online submission form.

Please remove any funding-related text from the manuscript and let us know how you would like to update your Funding Statement. Currently, your Funding Statement reads as follows:

This work was supported by the Medical Science and Technology Project of Zhejiang Province(No. 2022KY576,No.2023KY453).

Please include your amended statements within your cover letter; we will change the online submission form on your behalf.

A3: We have removed all funding-related text from the manuscript and revised the Funding Statement.

Q4. Thank you for stating the following in your Competing Interests section:

All authors disclosed no relevant relationships.

Please complete your Competing Interests on the online submission form to state any Competing Interests. If you have no competing interests, please state "The authors have declared that no competing interests exist.", as detailed online in our guide for authors at http://journals.plos.org/plosone/s/submit-now

This information should be included in your cover letter; we will change the online submission form on your behalf.

A4: We have added the statement "The authors have declared that no competing interests exist." to the Competing Interests section in the system.

Q5. Please note that your Data Availability Statement is currently missing the DOI/accession number of each dataset OR a direct link to access each database. If your manuscript is accepted for publication, you will be asked to provide these details on a very short timeline. We therefore suggest that you provide this information now, though we will not hold up the peer review process if you are unable.

A5: We have add the Data Availability Statement in the manuscript:

The data utilized in this study are publicly available at no cost from the National Health and Nutrition Examination Survey (NHANES) database (https://www.cdc.gov/nchs/nhanes/).

Reviewer #1:

Q1：Please clarify whether the study population is representative of the entire U.S. population or specific to a particular region within the country.

A1：The study population is drawn from the National Health and Nutrition Examination Survey (NHANES), which employs a complex, multistage probability sampling design to obtain a nationally representative sample of the civilian, non-institutionalized U.S. population. To ensure representativeness, we applied the recommended sampling weights, stratification, and clustering variables in all statistical analyses. We have added a clarification in the “Methods” section of the manuscript to reflect this point:

Page 4, Line 99: " Our data were sourced from the National Health and Nutrition Examination Survey (NHANES), a comprehensive and nationally representative health survey conducted by the Centers for Disease Control and Prevention (CDC) in the United States.”

Page 7, Line 36: " To generate nationally representative estimates, dietary weight was applied (wtdr2d).”

Q2：The study concludes a potential protective role of dietary anthocyanidins in reducing frailty, particularly among middle-aged individuals and males. Could the authors elaborate on why this effect is more pronounced in males compared to females, and why it appears significant in middle age but not in older adults?

A2: We have carefully revised the manuscript to provide further explanation of the observed age- and sex-specific associations.

Although frailty is more commonly observed in older adults, research suggests that many frailty-related physiological parameters begin to decline in middle-aged individuals [1]. Frailty is a dynamic and potentially reversible condition, and middle-aged adults have a higher likelihood of transitioning back to a robust state compared to older adults [2]. Therefore, this life stage may represent a critical window for early intervention. The significant inverse association observed between anthocyanidin intake and frailty in this age group may reflect the higher responsiveness of middle-aged individuals to dietary modulation.

In contrast, individuals under 40 years old typically experience peak physical function and lower baseline frailty risk, which may reduce the ability to detect a protective effect

of anthocyanidins in this group. Meanwhile, in adults aged 60 and above, the intake of anthocyanidins at or above the median level did not show a statistically significant reduction in frailty risk. One possible explanation is that older adults tend to have higher levels of oxidative stress and systemic inflammation[3]. These elevated levels of free radicals and inflammatory markers may exceed the antioxidant and anti-inflammatory capacity of dietary anthocyanidins, thus attenuating their protective effects in this population.

Additionally, sex-specific differences in the metabolism, bioavailability, and physiological response to flavonoids may contribute to the stronger association observed in males. For instance, males may have higher baseline oxidative stress or inflammatory burden[4], making them more susceptible to the beneficial effects of anthocyanidins. Hormonal differences, such as the modulating role of estrogen in females, may also influence the efficacy of flavonoid-related pathways[5].

We have incorporated these points into the "Discussion" section of the revised manuscript to improve clarity and interpretability:

Page 17 line 41:have been changed from：

"One plausible explanation for this observation is the presence of an excessive number of free radicals and inflammation markers, potentially overwhelming the antioxidant and anti-inflammatory capacities of anthocyanidins. Our subgroup analysis also revealed the significant between anthocyanidins consumption in male rather in female. The true reason is far from clear, and the difference in frailty transition between genders may be one of possible reasons."

to

"One possible explanation is that older adults tend to have higher levels of oxidative stress and systemic inflammation [41]. These elevated levels of free radicals and inflammatory markers may exceed the antioxidant and anti-inflammatory capacity of dietary anthocyanidins, thus attenuating their protective effects in this population. Our subgroup analysis also revealed the significant between anthocyanidins consumption in male rather in female. The true reason is far from clear, and the difference in the metabolism, bioavailability, and physiological response to flavonoids may contribute to the stronger association observed in males. For instance, males may have higher baseline oxidative stress or inflammatory burden [42], making them more susceptible

to the beneficial effects of anthocyanidins. Hormonal differences, such as the modulating role of estrogen in females, may also influence the efficacy of flavonoid-related pathways [43]."

Q3: As the authors mention, flavonoids are found in plant-based products such as tea, coffee, and wine. However, excessive consumption of these foods may not be beneficial. Is there a recommended daily intake of flavonoids for different age groups and genders?

A3：We agree that while flavonoid-rich foods such as tea, coffee, and red wine have been associated with various health benefits, their overconsumption may introduce health risks due to components such as caffeine, alcohol, or other bioactive compounds.

Currently, there is no established Recommended Dietary Allowance (RDA) or Adequate Intake (AI) level for total flavonoids or specific flavonoid subclasses, including anthocyanidins and flavanones, set by authoritative bodies such as the U.S. Institute of Medicine (IOM) or the European Food Safety Authority (EFSA). This is largely due to the variability in flavonoid content across food sources, differences in individual bioavailability and metabolism, and limited data from long-term randomized controlled trials.

Nevertheless, several observational studies suggest that a plant-based diet rich in fruits and vegetables, which naturally provides a wide range of flavonoids, is associated with favorable health outcomes, including reduced frailty risk. For example, intake levels of total flavonoids associated with health benefits in epidemiological studies typically range between 200–1000 mg/day, though these levels are not meant to serve as formal recommendations and can vary by population and dietary habits.

We have added a statement in the Discussion section to address this point: Page 17, Line 53:

"The ideal amount for reducing the frailty risk is an intake of 33.322 mg of total anthocyanidins per day."

Have been changed to

"Determining the optimal level of total anthocyanidin consumption is paramount, as both insufficient and excessive intake can result in nutritional imbalances. Currently, there is no officially established recommended daily intake for anthocyanidins. A previous observational study reported a U-shaped association between total flavonoid

intake and frailty, with the lowest risk observed at approximately 220 mg/day. In that study, the association between anthocyanidin intake and frailty was described as linear, making it difficult to identify a specific optimal dose [6]. However, it is important to note that the prior study focused solely on individuals aged 50 years and older, whereas our analysis included a broader adult population starting from 18 years of age. Based on our findings, the optimal intake of total anthocyanidins for reducing frailty risk is approximately 33.322 mg per day.”

Reviewer #2:

Introduction:

Q1: Regarding "Therefore, the aim of this study is to ……": Since the research has already been completed, it would be more appropriate to use the past tense.

A1: We have revised the sentence to past tense as suggested:

"Therefore, the aim of this study was to examine the association between six major anthocyanidins and total anthocyanidins consumption and frailty in a large and representative sample of US adults aged 18 years and older, using data from the National Health and Nutrition Examination Survey (NHANES) 2007-2010 and 2017-2018."

Q2: Authors claim that this is the first to investigate the link between anthocyanidin intake and frailty in a nationally representative U.S. adult population using NHANES data. However, a study published in 2025 in Frontiers in Nutrition analyzed data from NHANES 2007– 2010 and 2017–2018, and has examined the association between dietary flavonoid intake, including anthocyanidins, and frailty in U.S. adults. It would be beneficial to acknowledge and reference this prior work in the introduction to better contextualize the current study.

A2: We have revised the Introduction section of our manuscript and added this paper as citation 11. The updated text now reads (Page 3, Line 87):

"Previous research explored the relationship between dietary flavonoid intake and frailty in U.S. adults [10, 11]. However, these investigations excluded younger adults under the age of 20 and did not specifically assess the effects of individual anthocyanidin subtypes. Building upon this foundation, our study aimed to further explore the relationship between dietary anthocyanidin intake—including specific subtypes— and frailty in a nationally representative U.S. population."

Materials and Methods:

Q3: Why were only the NHANES cycles from 2007-2010 and 2017-2018 used, and not other years?

A3: The selection of NHANES cycles was based on the availability of dietary flavonoid intake data. Specifically, only the 2007–2008, 2009–2010, and 2017–2018 NHANES cycles included data on dietary flavonoid intake—including anthocyanidins—derived from the USDA Expanded Flavonoid Database, which was linked to the NHANES dietary recall data for those specific cycles.

Earlier or intermediate NHANES cycles did not provide comparable flavonoid data, and therefore could not be included in the analysis. We have added a sentence to the "Materials and Methods" section of the manuscript to clarify this point (Page 4, Line 5):

"We utilized de-identified secondary data from the NHANES cycles 2007–2010 and 2017–2018, as dietary flavonoid intake data were only available for these years. The combined dataset included 29,883 participants, of whom 17,888 were aged 18 years or older."

Q4: Figure numbers and figure titles with legends have not been included, appropriately.

Figures are not even placed in order, at the end of the manuscript.

A4: In the revised manuscript, we have carefully reviewed and corrected the figure formatting throughout.

Specifically:

- We have included figure numbers, titles, and detailed legends for all figures to ensure clarity and consistency.
- All figures have now been placed in the correct sequential order in the the manuscript.
- Furthermore, Figures 2, 3, and 4 have been consolidated into a single multi-panel figure labeled Fig 2a, 2b, and 2c, to improve clarity and to present the stratified analyses side by side for easier comparison.

Besides, it was the editor who requested us to remove all figures from the manuscript file. He said these would be automatically included in the reviewer' s PDF.

Q5: In Figure 1 (flow chart), it would be helpful to clearly mention the specific criterion/ criteria used for the initial exclusion of participants.

A5: We have revised the flow chart to explicitly state the criterion used for the initial exclusion. Specifically, we changed the label from:

“Excluding participants (N=11,995)”

to

“Excluding participants below 18 years old (N=11,995)”

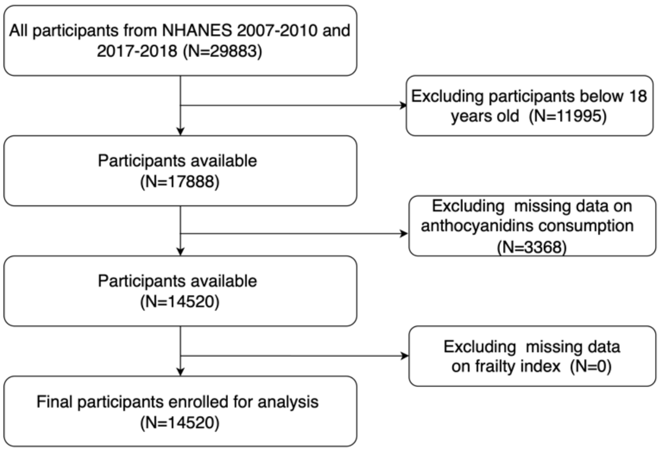

This revision more clearly communicates the basis for participant exclusion at the initial stage and enhances the transparency of our study design.

Q6: Why were only the first and second days of dietary intake used for analysis?

A6: In the present study, we utilized the average of the first and second 24-hour dietary recall data to estimate participants’ intake of anthocyanidins. This approach was based on the structure of the NHANES dietary data and the recommendations for estimating usual intake.

The NHANES dietary flavonoids database provides comprehensive flavonoid intake data derived from 24-hour dietary recalls, which are collected on two non-consecutive days for each participant. Using the average of both days helps to reduce within-person variation and better approximate the usual intake of flavonoids, including anthocyanidins. As such, we calculated the average intake of the six major anthocyanidins and total anthocyanidins based on these two days.

We have clarified this point in the revised Materials and Methods section with the following sentence (Page4, Line 23):

"To estimate usual intake more accurately and reduce day-to-day variability, the average anthocyanidin intake from the first and second 24-hour dietary recalls was calculated for each participant, as recommended for analyses using NHANES dietary data."

We appreciate the reviewer's suggestion, which helped us clarify this important methodological detail.

Q7: Regarding "To derive the frailty index value, the participant's acquired deficits' number is divided by the total potential deficits.": Suggest to cite correct primary source (reference) that authors used for Frailty Index Calculation.

A7: We have now included the correct primary sources for the calculation of the frailty index. The method we used for deriving the frailty index is based on the standard procedure outlined by Searle et al. (2008) *and Blodgett et al. (2015)*, which are widely adopted in frailty research.

We have updated the Materials and Methods section with the full citations:

- Searle SD, Mitnitski A, Gahbauer EA, Gill TM, Rockwood K. A standard procedure for creating a frailty index. BMC Geriatr. 2008;8:24. doi:10.1186/1471-2318-8-24.
- Blodgett J, Theou O, Kirkland S, Andreou P, Rockwood K. Frailty in NHANES: Comparing the frailty index and phenotype. Arch Gerontol Geriatr. 2015;60(3):464-470. doi:10.1016/j.archger.2015.01.016.

Results:

Q8: "Among these participants, 3,338 (18%) were non-frail, while 11,182 (82%) were classified as frail." Is this correct? Table 1 shows the opposite trends.

A8: Upon review, we found that the original statement was incorrect and inconsistent with the data presented in Table 1. As shown in Table 1, the majority of participants were classified as non-frail, while a smaller proportion were identified as frail.

We have corrected the sentence in the Results section to accurately reflect the data:

“Among these participants, 11,182 (82%) were non-frail, while 3,338 (18%) were classified as frail.”

This correction ensures consistency between the text and the data presented in the table.

Q9: Please recheck the calculated percentages presented in Table 1, as it appears discrepancies in the current values.

A9: We have carefully rechecked the calculations in Table 1. The percentages presented are derived from survey-weighted analyses using NHANES sampling weights, rather than unweighted counts. We have confirmed the accuracy of these weighted percentages and ensured that all values in the revised table are correct and appropriately reported.

Q10: In Table 1, “1N not Missing (unweighted), 2Mean ± SD; n (unweighted) (%), 3t-test adapted to complex survey samples; chi-squared test with Rao & Scott's second-order correction.” Here, numbers have not been superscripted.

A10: We have revised the numerical indicators in the footnotes of Table 1 to superscript format ([1], [2], [3]) to enhance clarity and conform to formatting conventions.

Under “Characteristics of the participants at baseline”:
Q11: It says “….. lower hemoglobin and uric acid levels, ……”, however, frail individuals exhibited higher uric acid levels. Please revise it to ensure correctness.

A11: We have corrected the description to accurately reflect the findings. The original sentence:

"They were also more likely to experience depressive symptoms, have higher white blood cell and platelet counts, lower hemoglobin and uric acid levels, higher creatinine and aspartate transaminase levels, lower alanine transaminase levels, and were burdened with comorbidities such as hypertension, hyperlipidemia, diabetes, stroke, or coronary heart disease."

has been revised to:

"They were also more likely to exhibit depressive symptoms; elevated white blood cell and platelet counts, uric acid, creatinine, and aspartate aminotransferase levels; reduced hemoglobin and alanine aminotransferase levels; and a higher prevalence of

comorbidities, including hypertension, hyperlipidemia, diabetes, stroke, and coronary heart disease."

Q12: Instead of "(refer to Table 1)", keep it as (Table 1).

A12: We have revised the text accordingly by replacing all instances of "(refer to Table 1)" with "(Table 1)" to align with standard scientific writing conventions.

Q13: Given the large sample sizes, there is a high likelihood of obtaining a statistically significant result even for small differences. Therefore, suggest to report the effect size (such as Cohen's d) alongside the p-value to assess the practical significance of the result.

A13: We agree that with large sample sizes, even small differences can yield statistically significant p-values. To address this, we have included effect size metrics alongside p-values to better reflect the practical significance of our findings. Specifically:

- For continuous variables, we computed Cohen's d (with unpooled standard deviations).
- For categorical variables, we reported Cramer's V.

These effect sizes are now displayed in a separate "Effect size" column in Table 1, which has been updated accordingly.

Q14: Additionally, based on the reported standard errors, it appears that the assumption of equal variances may not hold. Suggest "Levene's test" to formally assess the equality of variances. If the variances are unequal, the Welch's t-test, which does not assume equal variances, should be used instead of the standard Student's t-test.

A14: We performed Levene's test for all continuous variables to formally assess the assumption of homogeneity of variances across frailty groups. Based on the results of Levene's test, we selected the appropriate statistical test for group comparisons. Specifically, when the assumption of equal variances was met ($p \geq 0.05$), we applied the survey-weighted Student's t-test; otherwise ($p < 0.05$), the survey-weighted Welch's t-test was used. The test selection for each variable is now documented in the updated Table 1, and the corresponding statistical method has been explicitly stated in the footnotes. These revisions ensure that variance heterogeneity is properly accounted for, thereby improving the robustness of our comparisons.

Q15: For comparisons using the chi-square test, since the sample size is large, the assumption regarding expected frequencies is likely satisfied. However, when working with contingency tables larger than 2×2, and if the result is statistically significant, it is important to perform a standardized residual analysis. This helps to identify which specific cells (i.e., combinations of categories) contribute most to the observed association.

A15: We have revised and expanded the "Statistical Analyses" section to address the concerns raised. Specifically, we:

- Provided detailed descriptions of the statistical tests used to compare participant characteristics across frailty status, based on variable type and variance assumptions.
- Conducted Levene's test to assess the homogeneity of variances for all continuous variables and applied either survey-weighted Student's t-tests or survey-weighted Welch's t-tests accordingly.
- For categorical variables, we used survey-weighted Rao–Scott corrected chi-square tests.
- In addition to reporting p-values, we included effect sizes to reflect the practical significance of group differences: Cohen's d for continuous variables and Cramer's V for categorical variables.
- Moreover, for categorical variables with significant associations ($p < 0.05$) and more than two levels, we performed standardized residual analyses to identify cells contributing most to the overall chi-square statistic.

The revised text is highlighted in yellow in the Statistical Analyses section of the manuscript to facilitate review.

Q16: Consider removing the full-stop at the end of subheadings.

A16: In accordance with the suggestion, we have removed the full stops at the end of all subheadings throughout the manuscript to ensure consistency with PLOS ONE formatting guidelines.

Q17: Under "Relationship between anthocyanidins consumption and frailty": consider adding the word "respectively" in relevant sentences to clarify the order of items and improve readability.

A17: To enhance clarity and improve readability, we have added the word "respectively" in the relevant sentences to clarify the correspondence between the odds ratios and the respective models. The revised paragraph now reads:

Revised Text (with "respectively" added)(Page 10 Line 74,75,78,81):

Table 2 illustrates the association between specific anthocyanidins and total anthocyanidins intake with frailty. Increased consumption of malvidin, delphinidin, and total anthocyanidins was significantly and inversely linked to the odds ratio of frailty across all models. Participants with malvidin intake-above the median exhibited ORs of 0.64 (95% CI: 0.55-0.75) and 0.49 (95% CI: 0.42-0.57) for frailty in the crude and Model 1, respectively, and ORs of 0.69 (95% CI: 0.53-0.88) in Model 2 and 0.67 (95% CI: 0.49-0.92) in Model 3, respectively. Similarly, participants with above median delphinidin consumption showed ORs of 0.62 (95% CI: 0.54-0.73) in the crude model, 0.48 (95% CI: 0.41-0.57) in Model 1, 0.74 (95% CI: 0.57-0.96) in Model 2, and 0.73 (95% CI: 0.54-0.99) in Model 3, respectively. Additionally, the OR for above-median total anthocyanidins intake in the crude model was 0.6 (95% CI: 0.48-0.74), decreasing to 0.41 (95% CI: 0.32-0.51) in Model 1, and rising to 0.61 (95% CI: 0.44-0.85) and 0.59 (95% CI: 0.39-0.90) in Models 2 and 3, respectively.

Q18: "Similarly, the overall relationship between different types of anthocyanidins and frailty was not significant in all models, except for delphinidin, which exhibited an OR of 0.97 (95% CI: 0.96, 0.99), 0.96 (95% CI: 0.94,0.98), 0.96 (95% CI: 0.94, 0.99), and 0.97 (95% CI: 0.94, 1.00) in the crude model, Model 1, Model 2, and Model 3, respectively." Check this for clarity. Is this consistent with Table 2?

A18: We acknowledge that the original sentence was misleading due to a writing error. The phrase "the overall relationship … was not significant in all models" was inaccurate and potentially confusing. To improve clarity and consistency with Table 2, we have revised the sentence as follows:

Original sentence (incorrect):

"Similarly, the overall relationship between different types of anthocyanidins and frailty was not significant in all models, except for delphinidin, which exhibited an OR of 0.97 (95% CI: 0.96, 0.99), 0.96 (95% CI: 0.94, 0.98), 0.96 (95% CI: 0.94, 0.99), and 0.97 (95% CI: 0.94, 1.00) in the crude model, Model 1, Model 2, and Model 3, respectively."

Revised sentence (corrected for clarity):

"Similarly, the overall relationship between different types of anthocyanidins and frailty was not significant, except for delphinidin, which exhibited an OR of 0.97 (95% CI: 0.96, 0.99), 0.96 (95% CI: 0.94, 0.98), 0.96 (95% CI: 0.94, 0.99), and 0.97 (95% CI: 0.94, 1.00) in the crude model, Model 1, Model 2, and Model 3, respectively."

This correction aligns the text more accurately with the findings presented in Table 2.

Q19: In table 2, "a, b, c, and d" are not superscripted.

A19: We have corrected this formatting issue by superscripting the letters "a," "b," "c," and "d" in Table 2 to ensure proper referencing and consistency with journal formatting guidelines.

Q20: In table 2, "overall c": what "c" stands for?

A20: This was a typo mistake. We have corrected "overall c" to "overall e" in Table 2. The superscript "e" refers to the analysis of each type of anthocyanidin and total anthocyanidins as continuous intake variables.

Q21: In table 2: "cModel 3 was additionally adjusted for white blood cells count (1000 cells/ul), …….. alanine transaminase (u/l) and aspartate transaminase, hypertension, …….., and stroke." Here, the unit for aspartate transaminase is missing, while units are clearly stated for other parameters. Suggest to include the appropriate units for consistency and clarity.

A21: We agree with your comment and have now added the missing unit for aspartate transaminase to ensure consistency and clarity in the reporting of biochemical parameters. The revised footnote has been updated accordingly.

Revised Table 2 Footnote (with units standardized)

Abbreviations: OR, odds ratio; CI, confidence interval.

a Model 1 was adjusted for age, sex (male and female) and race (Mexican American, non-Hispanic black, non-Hispanic white, other races).

b Model 2 was additionally adjusted for education (below high school, high school, above high school), marital status (married/living with partner, never married,

divorced/separated, widowed), poverty-income ratio (continuous), body mass index （BMI） (<18.5, 18.5≤BMI<25, 25≤BMI<30, ≥30, kg/m2), drinking status (no drinker, moderate drinker, heavy drinker), smoking (never, former, current), caffeine consumption(continuous, g/day), total dietary energy intake(continuous, kcal/day), and physical activity(low, moderate, high);

c Model 3 was additionally adjusted for white blood cells count (1000 cells/μL), hemoglobin (g/dL), platelets count (1000 cells/μL), albumin (g/dL), creatinine (mg/dL), uric acid (mg/dL), alanine transaminase (μ/L) and aspartate transaminase (μ/L), hypertension, hyperlipidemia, depressive symptom, diabetes, coronary heart disease, and stroke.

D The consumption of each type of anthocyanidin was categorized into three groups: Group 1 (no consumption, i.e., intake = 0), Group 2 (intake below the median), and Group 3 (intake at or above the median).

e Analysis of each anthocyanidin subtype and total anthocyanidins as continuous intake variables.

Under "subgroup analysis of the relationship between anthocyanidins consumption and frailty",
Q22: It is mentioned that "In the 41-60 age group, an elevated intake of malvidin at or above the median correlated with a decreased frailty risk (OR = 0.769; 95% CI: 0.563-1.051)." However, OR and CI values do not appear to correspond correctly with those in Table 3. Please review and revise accordingly.

A22: We have reviewed the values and acknowledge the discrepancy between the reported odds ratio and the data in Table 3. The sentence has now been corrected to:

"In the 41-60 age group, an elevated intake of malvidin at or above the median correlated with a decreased frailty risk (OR = 0.514; 95% CI: 0.359 - 0.735)."

Q23: Regarding "Similarly, the odds ratio of frailty was 0.567 (95% CI: 0.404-0.796) for consuming less than the median of total anthocyanidins, decreasing further to 0.452 (95% CI: 0.318-0.644) in 41-60 years old adults.": Here, it appears to refer to Group 3, however, this is not clearly stated. Please clarify.

A23: We have revised the sentence accordingly for improved clarity. The updated version now reads:

"Similarly, the odds ratio of frailty was 0.567 (95% CI: 0.404–0.796) for participants consuming less than the median of total anthocyanidins, and decreased further to 0.452 (95% CI: 0.318–0.644) among adults aged 41–60 years whose intake was at or above the median."

Q24: Regarding "Specifically, higher intake of malvidin, petunidin, and delphinidin significantly correlated with lower odds of frailty in males, with odds ratios (OR) of 0.724 (95% CI: 0.525-0.998), 0.673 (95% CI: 0.491-0.923), and 0.702 (95% CI: 0.519-0.950), respectively.": Here, is higher intake of malvidin significantly correlated with lower odds of frailty in males, as p value is 0.055? In methodology, the authors stated that the significance level was set at 0.05. Please justify the rationale.

A24: Regarding the statement *"Specifically, higher intake of malvidin, petunidin, and delphinidin significantly correlated with lower odds of frailty in males…"*, we acknowledge the reviewer's concern about the p-value of 0.055 for malvidin. However, we would like to clarify that the p-value referenced in Table 3 for malvidin in males corresponds to the P for trend, not the p-value for the group comparison itself.

In statistical analysis, P for trend is used to assess whether there is a statistically significant dose–response relationship across ordered categories of exposure—in this case, increasing levels of anthocyanidin intake. It tests the hypothesis that the outcome variable changes in a consistent direction as the exposure level increases. Thus, it does not directly assess the significance of the effect in a specific subgroup.

The odds ratio and 95% confidence interval provided for malvidin (OR = 0.724, 95% CI: 0.525–0.998) indicate a statistically significant association at the conventional 0.05 threshold, as the confidence interval does not include 1. Therefore, the conclusion that higher intake of malvidin is significantly associated with lower odds of frailty in males remains statistically valid.

Q25: In table 3, what are meant by "P for trend" and "P for interaction"?

A25: In Table 3, the terms are defined as follows:

- P for trend evaluates whether there is a linear trend across ordered categories of anthocyanidin intake (e.g., from non-consumption to high consumption), reflecting a potential dose–response relationship.
- P for interaction assesses whether the association between anthocyanidin intake and frailty differs significantly between subgroups (e.g., by sex or age group).

A statistically significant interaction suggests that the effect of anthocyanidin intake on frailty risk is modified by the subgroup factor.

Q26: Suggest to indicate how the p-values were generated (e.g., statistical test used) in the footnote of Table 3 for clarity.

A26: We have added an explanatory note in the footnote of Table 3 to clarify how the p-values were generated. Specifically:

- The p-values for trend were calculated using *survey-weighted logistic regression models* treating the median-based intake categories of each anthocyanidin as an ordinal variable (0 = non-consumer, 1 = below-median, 2 = above-median). This approach tests for a dose–response relationship across increasing levels of anthocyanidin intake, accounting for the complex sampling design of NHANES.
- The p-values for interaction were derived from *survey-weighted logistic regression models* that included an interaction term between anthocyanidin intake and the subgroup variable (e.g., sex or age group). A significant p-value (typically <0.05) indicates that the effect of anthocyanidin intake on frailty differs significantly across the levels of the subgroup factor.

We have revised the footnote of Table 3 accordingly to improve clarity and transparency for readers.

Updated table footnote:

"P for trend was derived from survey-weighted logistic regression models treating intake category as an ordinal variable. P for interaction was obtained from models including a cross-product term between anthocyanidin intake and the subgroup variable, accounting for NHANES complex survey design."

Q27: In table 3, "a" (at the end of group names) needs to be superscripted.

A27: We have revised Table 3 to ensure that all footnote indicators, including the letter "a" at the end of group names, are properly formatted as superscripts to align with academic and journal formatting standards.

Q28: Authors have used four logistic regression models to assess the association between anthocyanidin intake and frailty. However, a formal comparison of model fit,

such as a likelihood ratio test (using ANOVA on nested models) or reporting AIC/BIC values can be performed to justify the incremental addition of covariates across models. It would strengthen the rationale for model selection and highlight whether each adjustment meaningfully improves explanatory power. Since your focus is on model-adjusted associations, demonstrating improvements in model fit could further support the robustness of your conclusions.

A28: We agree that formally assessing model fit would strengthen the rationale for the incremental inclusion of covariates.

As suggested, we initially explored the use of likelihood ratio tests or information criteria (AIC/BIC) to compare nested models. However, because the analysis is based on data from the National Health and Nutrition Examination Survey (NHANES) with a complex sampling design, and the models were fitted using the svyglm() function from the survey package in R, these metrics are not directly available. Specifically, svyglm() does not use full maximum likelihood estimation but rather quasi-likelihood methods, making likelihood-based comparisons such as AIC, BIC, or likelihood ratio tests technically inappropriate. Attempting to extract logLik or AIC from svyglm models results in warnings indicating these methods are not valid in this context.

To address this limitation, we employed McFadden's Pseudo R^2 as an approximate measure of model fit. Although not a direct analog of the R^2 used in linear regression, McFadden's Pseudo R^2 is widely used for evaluating logistic regression models. It is calculated as:

Pseudo R^2 = 1 - (Residual Deviance of Full Model / Residual Deviance of Null Model)

This statistic provides a sense of how well the model explains the variation in the outcome variable compared to a null model. The interpretative guideline for McFadden's Pseudo R^2 values is as follows:

- < 0.1: Poor fit
- 0.1 – 0.3: Modest fit
- 0.3 – 0.5: Moderate fit
- > 0.5: Strong model fit

We applied this method to evaluate the model fit for the four sequential logistic regression models analyzing the association between anthocyanidin intake and frailty. Below we present the example of total anthocyanidin intake:

The calculated McFadden Pseudo R^2 values were: Model 1: 0.092, Model 2: 0.562, Model 3: 0.695. These results indicate a modest improvement from the unadjusted to Model 1 (from 0 to 0.092), followed by a substantial increase in explanatory power with the inclusion of lifestyle, laboratory, and clinical variables in Models 2 and 3. The final model (Model 3) demonstrates a strong model fit, explaining approximately 69.5% of the deviance relative to the null model. Similar improvements in model fit were observed for each anthocyanidin subtype (e.g., cyanidin, delphinidin, malvidin), with consistent patterns supporting the robustness of our findings.

We have now included these details in the revised manuscript, including a description of the Pseudo R^2 method in the Statistical Analysis section and model fit values in the Results section.

Thank you again for your insightful suggestion, which helped us strengthen the justification for our model selection approach.

Q29: Supplementary Tables have not been attached along with the manuscript.

A29: Thank you for your kind notice. Supplementary Tables has now been uploaded along with the revised manuscript. In addition, we have recalculated all relevant results presented in the supplementary table and updated the corresponding description in the manuscript accordingly. The revised text in the Results section now reads:

"Interestingly, a significant inverse association with frailty was observed for higher (above-median) intake of malvidin, petunidin, delphinidin, and total anthocyanidins, whereas no such association was found for cyanidin, peonidin, or pelargonidin. Furthermore, in the fully adjusted models examining the overall association between individual anthocyanidins and frailty, significant relationships persisted for petunidin and delphinidin (S2 Table)."

Q30: Under “Dose-response relationships between anthocyanidins intake and frailty risk”: it says “In the investigation of ….. total anthocyanidins intake and frailty ($P < 0.01$). is the p value mentioned, correct?

A30: The correct value should be $P < 0.001$, not $P < 0.01$. We have corrected this in the revised manuscript accordingly.

Discussion:

Q31: Fourth paragraph: ("our study exhibits …….. progression over time.") appears to be paraphrased again in the fifth paragraph.

A31: The overlapping content has been removed, and we have retained only one consolidated paragraph to avoid repetition and improve clarity.

Q32: Suggest to incorporate findings from a similar recent study published in 2025 in Frontiers in Nutrition to enrich your discussion and provide up-to-date context.

A32: We have incorporated findings from the recent study by Cai et al. (2025) published in *Frontiers in Nutrition* [Cai et al., 2025. *Front Nutr.* 12:1490998], as well as the related work by Guo et al. (2024) published in *The Journal of Frailty & Aging* [Guo et al., 2024. *J Frailty Aging*, 13(4):359–368]. These studies have been referenced and integrated into the revised Discussion section to enrich the context and provide a more up-to-date perspective on the association between dietary flavonoid intake and frailty risk. The discussion has also been expanded to highlight the consistency and distinctions between our findings and those of prior research.

References:

Q33: Review and revise the references to ensure consistency in formatting throughout the manuscript.

A33: We have carefully reviewed and revised all references in the manuscript to ensure consistency in formatting according to the journal's guidelines. This includes standardizing author names, journal titles, volume and issue numbers, page ranges, publication years, and DOI formatting throughout the reference list.

Overall/ common:

Q34: Fonts and font sizes are not uniform throughout the manuscript.

A34: We have thoroughly reviewed the manuscript and corrected all inconsistencies in fonts and font sizes to ensure a uniform and professional appearance throughout the document, in accordance with the journal's formatting requirements.
