# Supplementary material for: The inverse relationship between dietary anthocyanidins consumption and frailty: Findings from the National Health and Nutrition Examination Survey
Source: PLoS One. 2025 Aug 1;20(8):e0328489. doi: 10.1371/journal.pone.0328489 (PMC12316302; doi:10.1371/journal.pone.0328489)
Supplement: Attachment — Submitted filename: Comments_R_PONE-D-25-11030_R1- 27.06.2025.pdf [file pone.0328489.s005.pdf]

Reviewer’s Comments

Title: The inverse relationship between dietary anthocyanidins consumption and Frailty: Findings from the National Health and Nutrition Examination Survey

I appreciate authors’ efforts in revising the manuscript. Most of the comments have been addressed satisfactorily, though a few minor points still require attention.

Materials and Methods:

- Regarding “To derive the frailty index value, the participant's acquired deficits' number is divided by the total potential deficits.”: According to previously suggestion, authors have cited the primary sources for Frailty Index Calculation. Kindly check whether those are cited in the correct place.

Results:

- Regarding Table 1:
    i. N and n should be defined at the bottom of table 1.
    ii. Authors have stated that the presented percentages are based on survey-weighted analyses using NHANES sampling weights. To enhance transparency and interpretability for readers, it may be helpful to also report the weighted sample size. Including both the unweighted and weighted numbers, either in the table or as a footnote, would provide clarity regarding the findings.
    iii. The authors have included effect size metrics as suggested. It is appreciated. However, they have not provided any interpretation of these metrics, in terms of their relevance within the results section.
- Regarding Table 2: The letter 'e' in the table footnote should be bolded for consistency with the formatting of other footnote indicators.
- Although the authors have mentioned the supplementary tables, these have not yet been attached to the manuscript.

References:

- A few references are still not formatted uniformly. Please review and revise the reference list to ensure consistency in formatting, adhering to the required citation style.
